# Supplementary material for: A Porphyrin-Based Covalent Organic Framework as Metal-Free Visible-LED-Light Photocatalyst for One-Pot Tandem Benzyl Alcohol Oxidation/Knoevenagel Condensation
Source: Nanomaterials (Basel). 2023 Jan 30;13(3):558. doi: 10.3390/nano13030558 (PMC9920377; doi:10.3390/nano13030558)
Supplement: Supplementary file 1 [file nanomaterials-13-00558-s001.zip › nanomaterials-2177967-supplementary.pdf]

Supporting Information

A porphyrin-based covalent organic framework as metal-free visible-LED-light photocatalyst for one-pot tandem benzyl alcohol oxidation/Knoevenagel condensation

Sara Oudi,[1] Ali Reza Oveisi,[1,*] Saba Daliran,[1] Mostafa Khajeh,[1] Amarajothi Dhakshinamoorthy,[2,3] Hermenegildo García[2,*]

[1] Department of Chemistry, Faculty of Sciences, University of Zabol, P.O. Box: 98615-538, Zabol, Iran

[2] Departamento de Quimica, Universitat Politècnica de València, Av. De los Naranjos s/n, 46022 Valencia, Spain

[3] School of Chemistry, Madurai Kamaraj University, Madurai 625021, India

* Correspondence: aroveisi@uoz.ac.ir or alir.oveisi@gmail.com (A. R. Oveisi), hgarcia@qim.upv.es (H. Garcia)

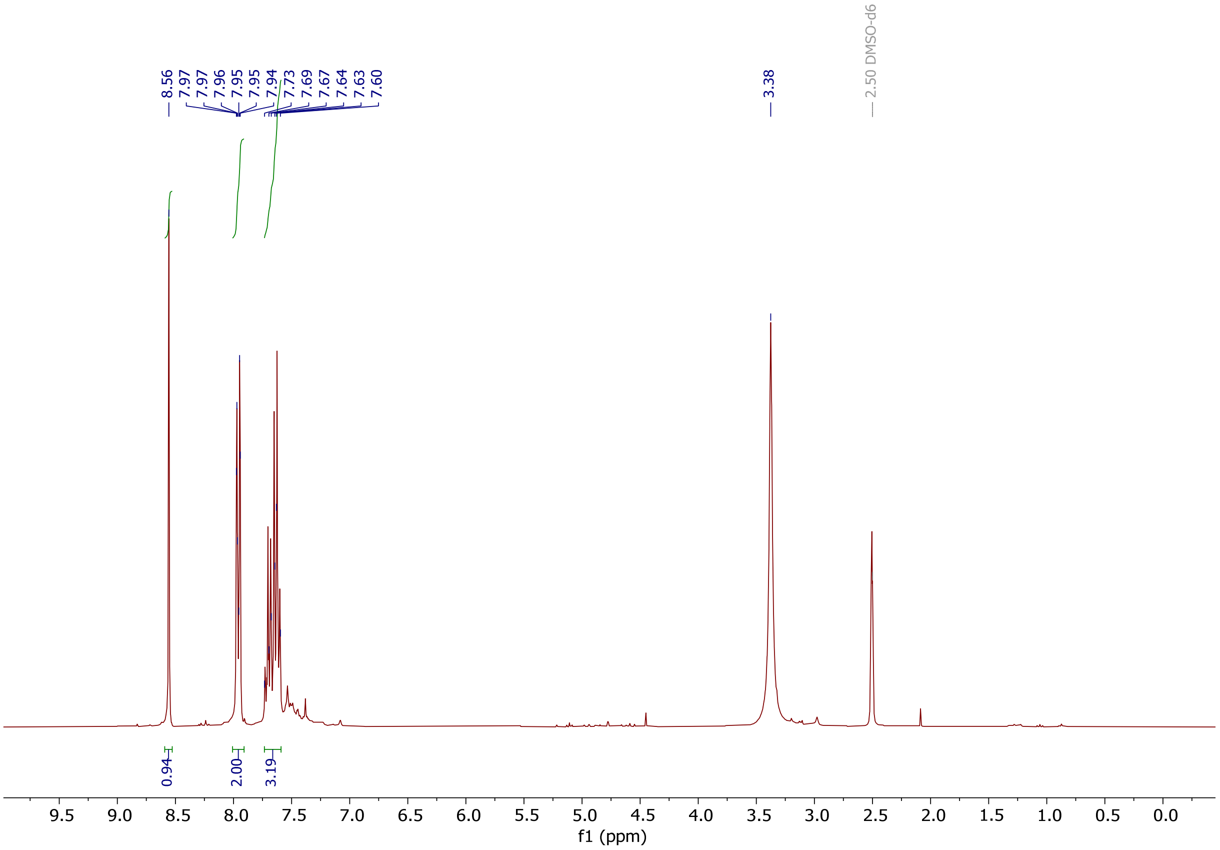

Figure S1. ^{1}H NMR spectrum of 2-benzylidenemalononitrile.

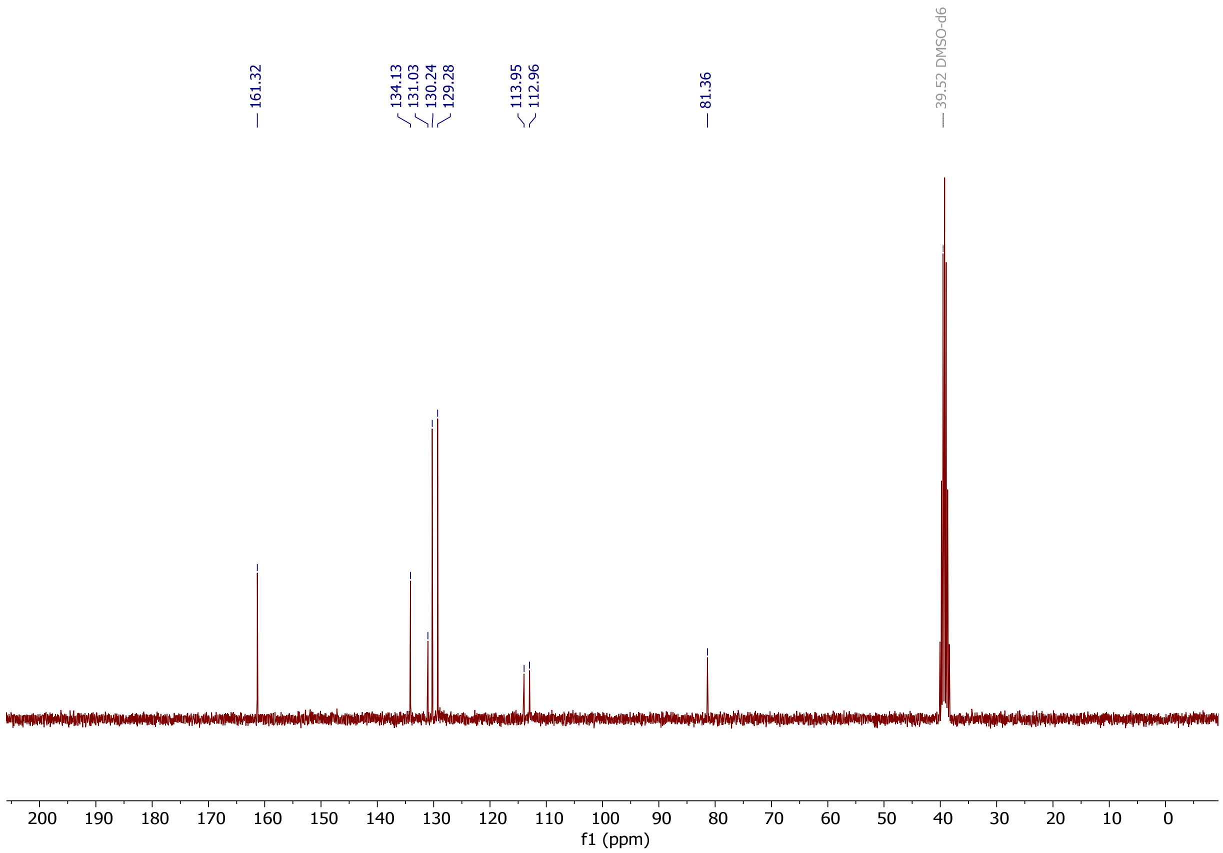

Figure S2. ^{13}C NMR spectrum of 2-benzylidenemalononitrile.

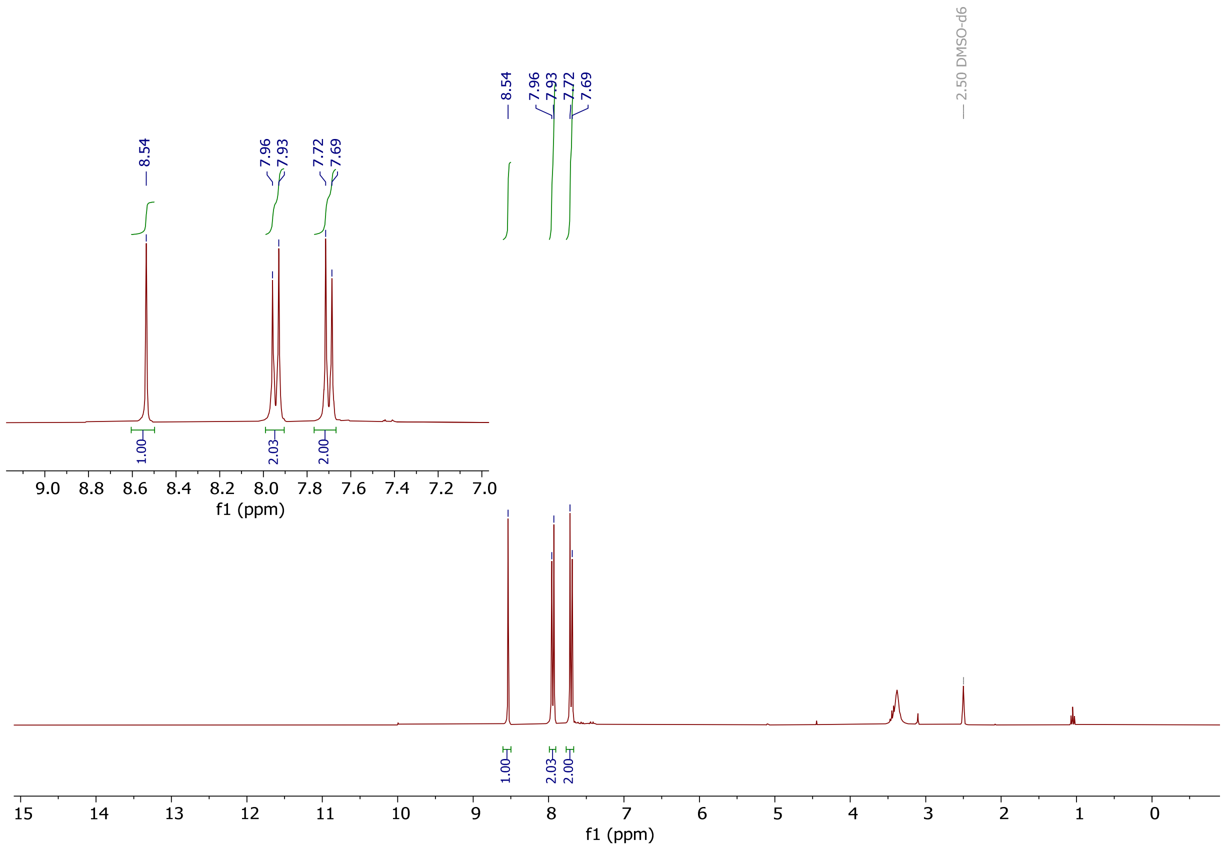

Figure S3. ^{1}H NMR spectrum of 2-(4-chlorobenzylidine)malononitrile.

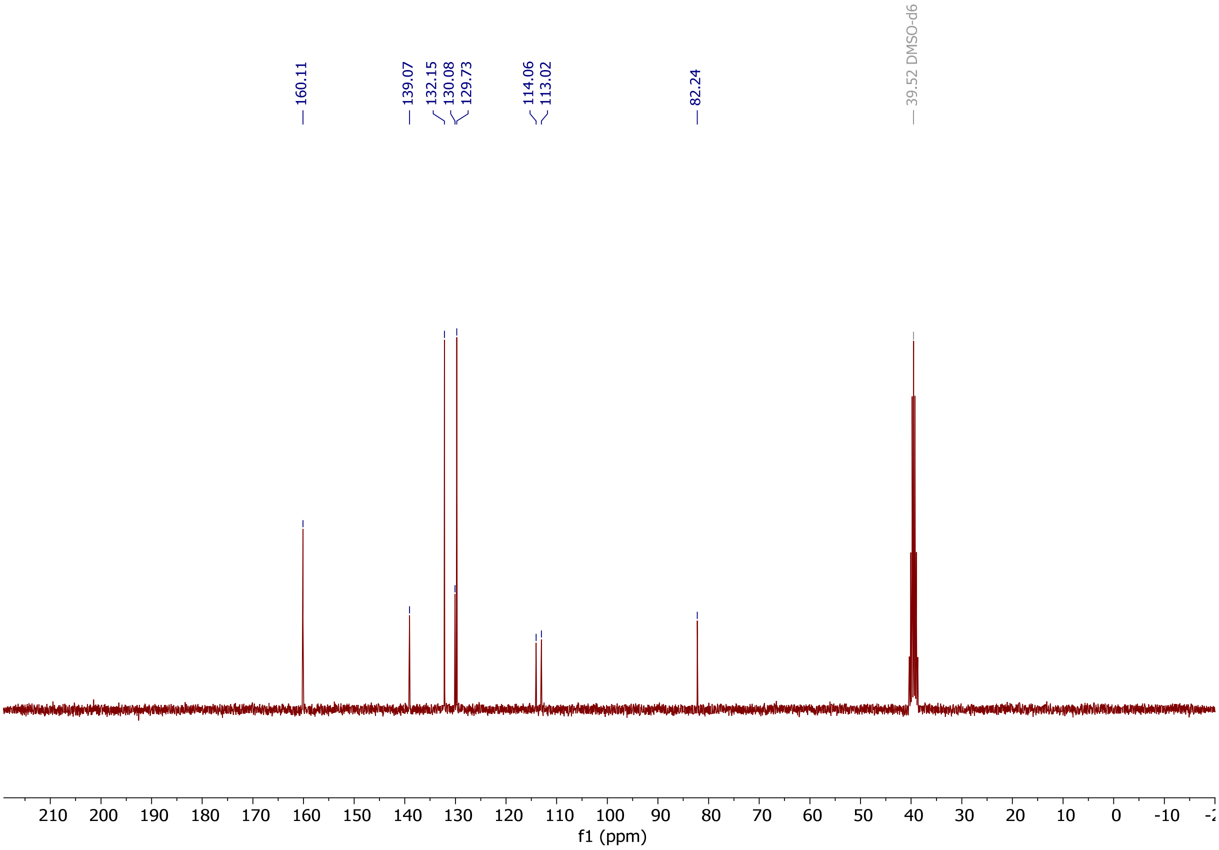

Figure S4. ^{13}C NMR spectrum of 2-(4-chlorobenzylidine)malononitrile.

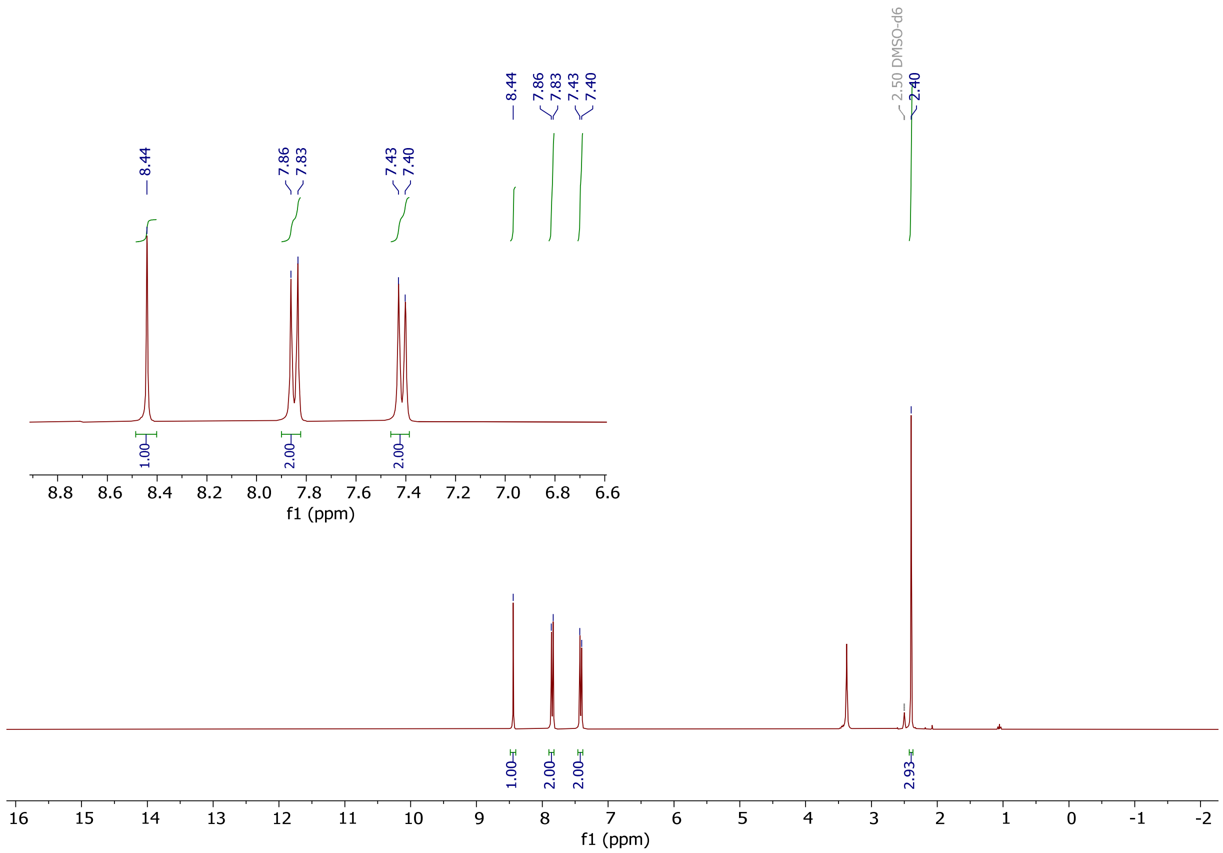

Figure S5. ^{1}H NMR spectrum of 2-(4-methylbenzylidene)malononitrile.

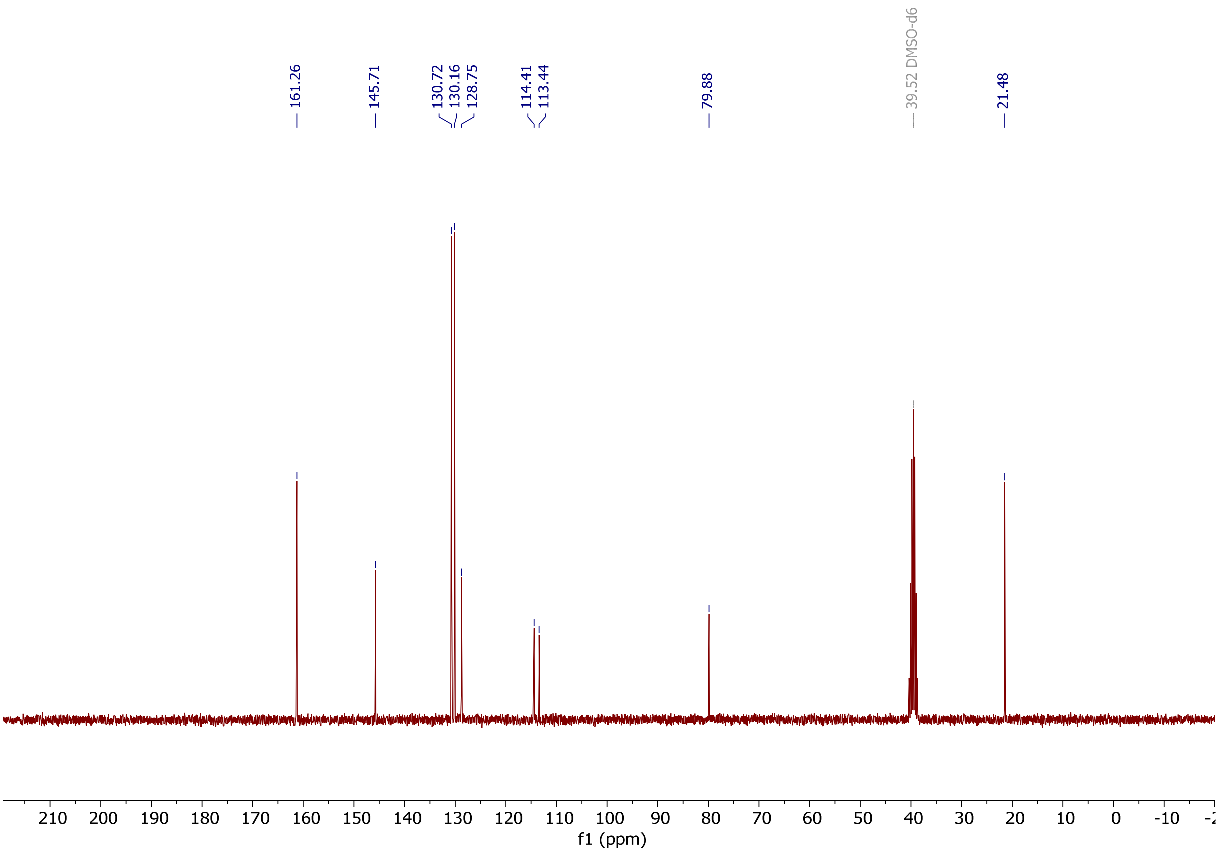

Figure S6. ^{13}C NMR spectrum of 2-(4-methylbenzylidene)malononitrile.

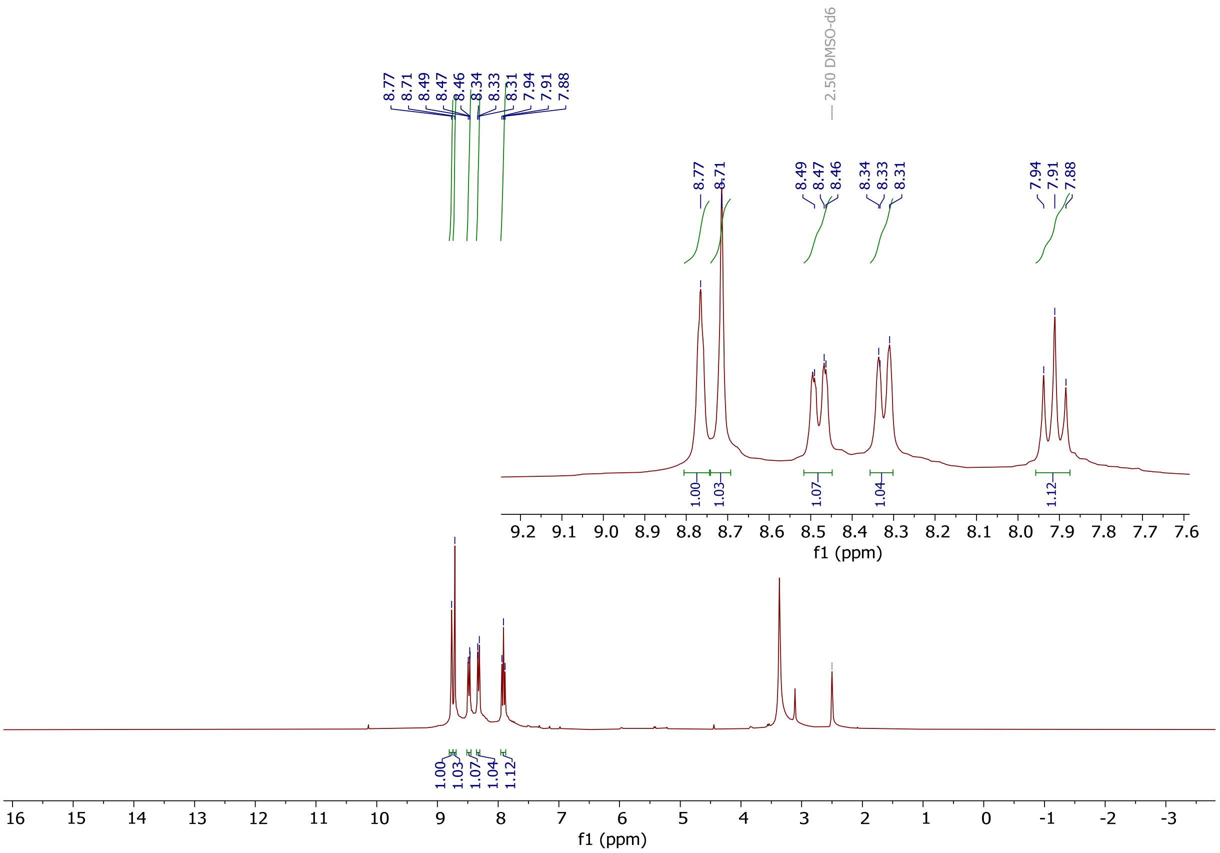

Figure S7. ^{1}H NMR spectrum of 2-(3-nitrobenzylidene)malononitrile.

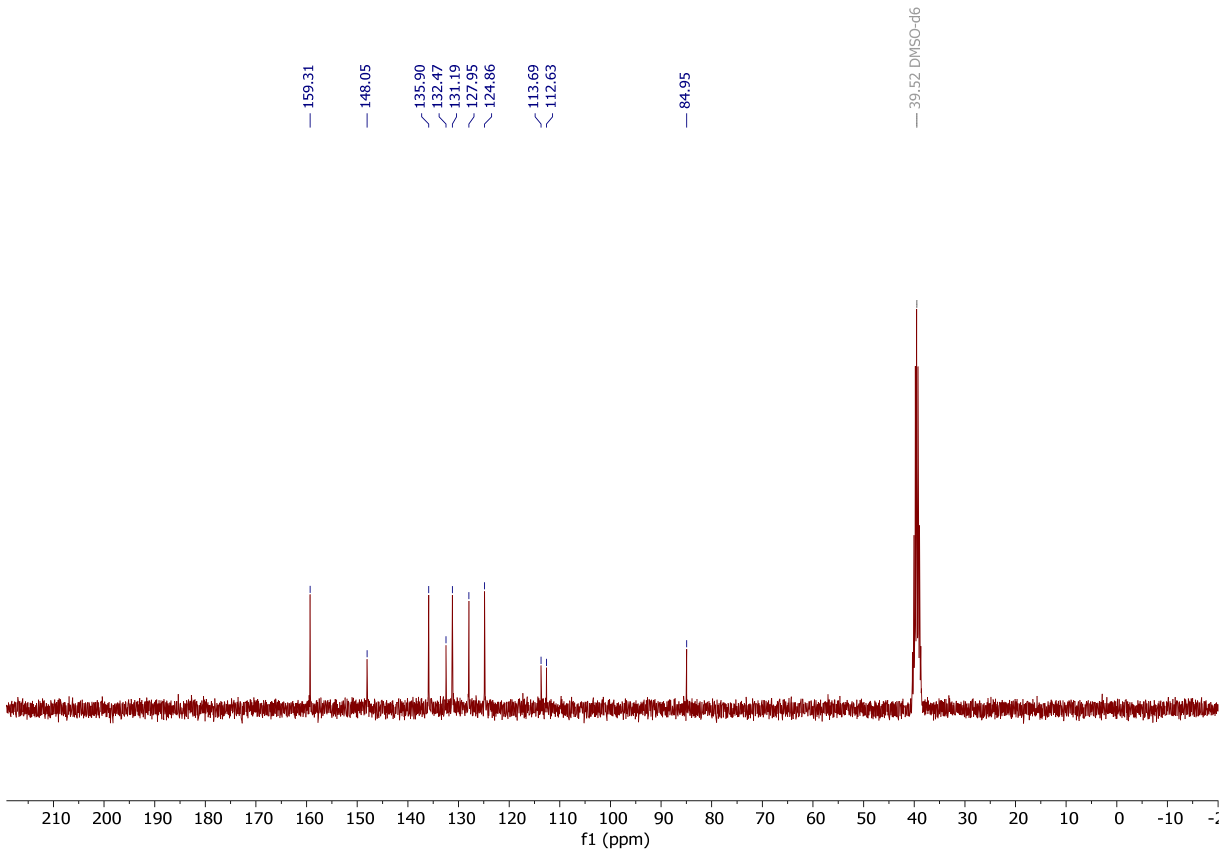

Figure S8. ^{13}C NMR spectrum of 2-(3-nitrobenzylidene)malononitrile.

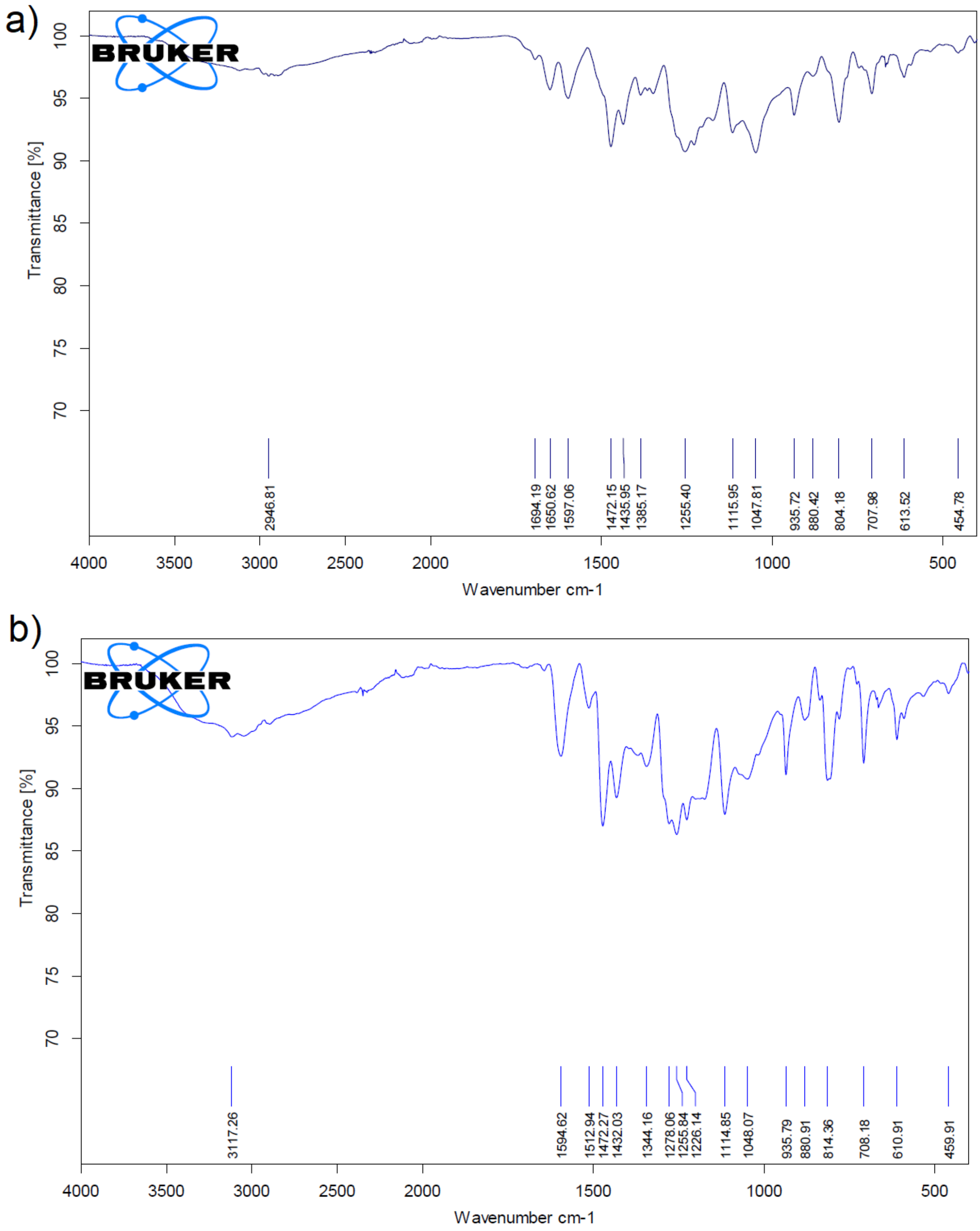

Figure S9. FT-IR spectra of fresh (a) and reused (b) Porph-UOZ-COF.

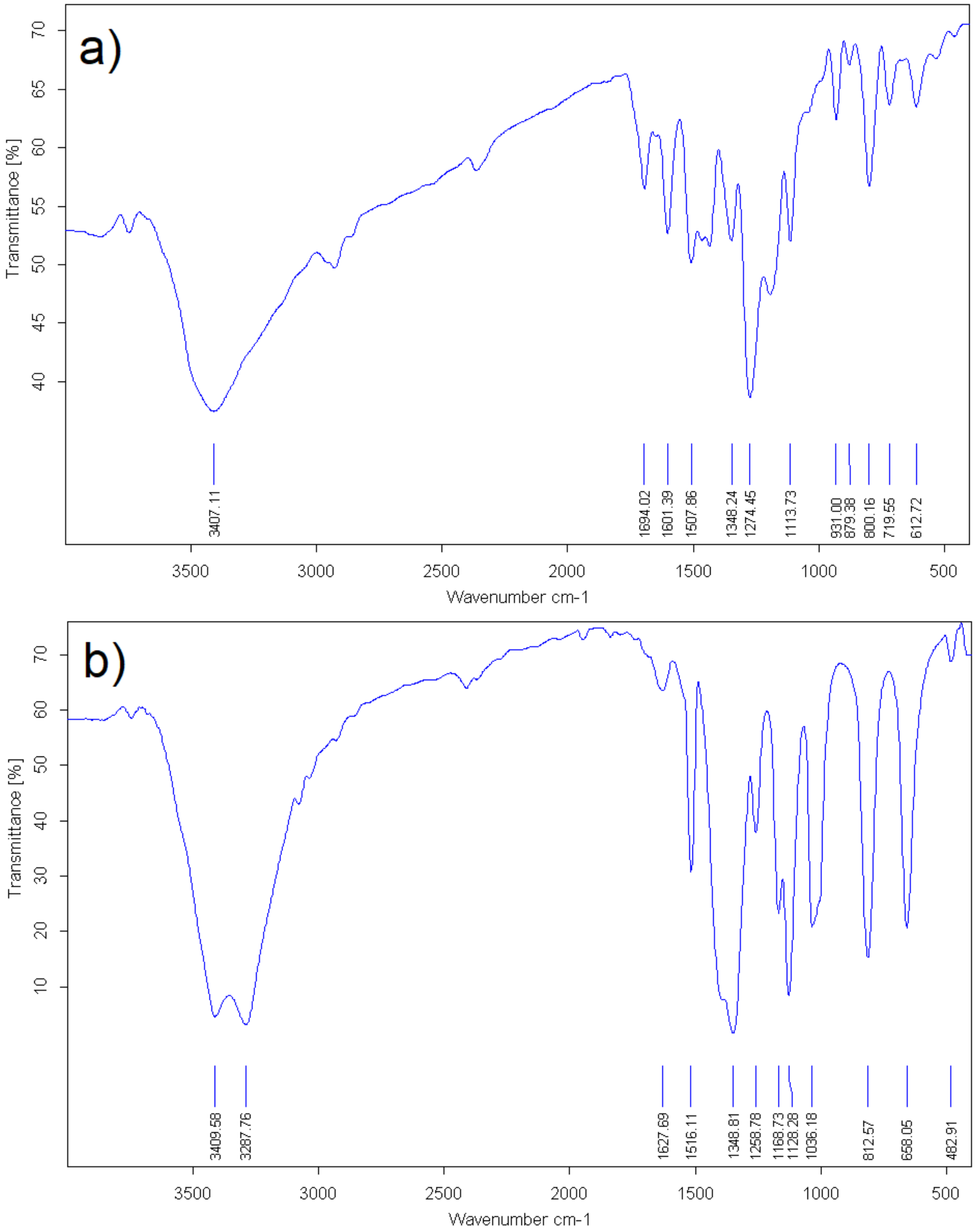

Figure S10. FT-IR spectra of monomers: a) DHPP and b) DBBA.

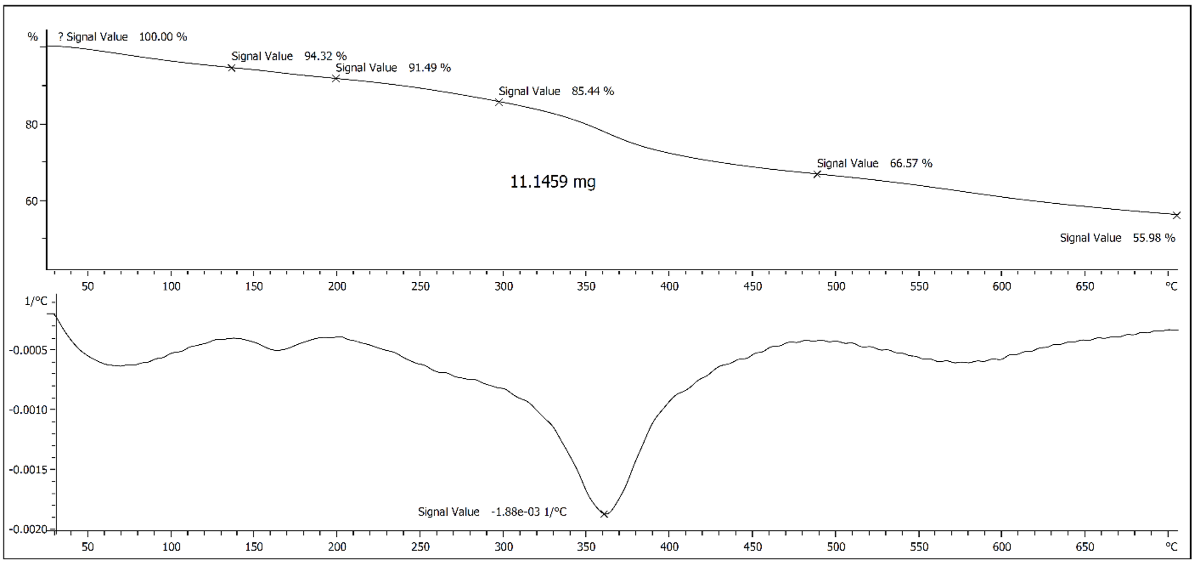

Figure S11. TGA/DSC profile of the COF.

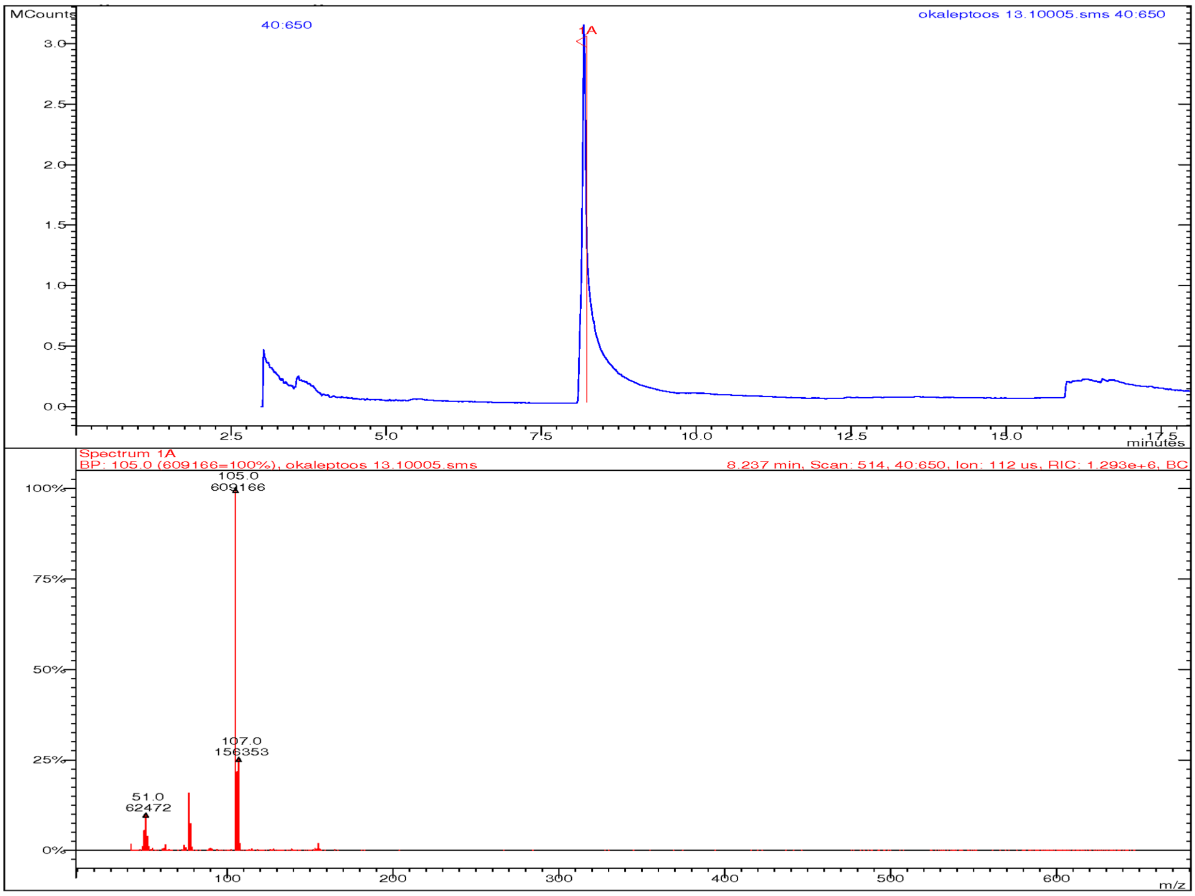

Figure S12. GC/MS spectrum of benzaldehyde as the intermediate (retention time of ~ 7.8).

Product characterizations:

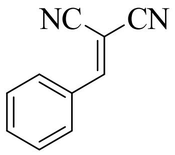

2-Benzylidenemalononitrile

Orange Solid, 82-84 °C (81-83°C).[S1] ^{1}H NMR (300 MHz, DMSO-d_6) δ ppm: 8.56 (s, 1H), 7.97-7.95 (m, 2H), 7.73-7.60 (m, 2H). ^{13}C NMR (75 MHz, DMSO-d_6) δ ppm: 161.32, 134.13, 131.0, 130.2, 129.2, 113.9, 112.9, 81.3.

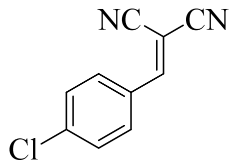

2-(4-Chlorobenzylidine)malononitrile

Yellow Solid, 160-162 °C (160-162°C).[S1] IR (KBr): 3445, 2224, 1581, 1478, 1216, 1094 cm^{-1}. ^{1}H NMR (300 MHz, DMSO-d_6) δ ppm: 8.54 (s, 1H), 7.94 (d, J = 9 Hz, 2H), 7.70 (d, J = 9 Hz, 2H). ^{13}C NMR (75 MHz, DMSO-d_6) δ ppm: 160.1, 139, 132.1, 130, 129.7, 114, 113, 82.2.

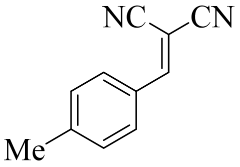

2-(4-Methylbenzylidene)malononitrile

Brown Solid, 132-135 °C (132-134°C).[S2] IR (KBr): 3444, 2226, 1524, 1347, 1213 cm^{-1}. ^{1}H NMR (300 MHz, DMSO-d_6) δ ppm: 8.44 (s, 1H), 7.85 (d, J = 9 Hz, 2H), 7.42 (d, J = 9 Hz, 2H), 2.40 (s, 3H). ^{13}C NMR (75 MHz, DMSO-d_6) δ ppm: 161.2, 145.7, 130.7, 130.1, 128.7, 114.4, 113.4, 79.8, 21.4.

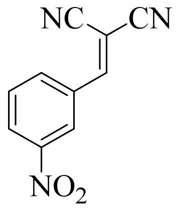

2-(3-Nitrobenzylidene)malononitrile

Orange Solid, 102-104 °C (98-101° C).[S3] IR (KBr): 3444, 2219, 1585, 1186 cm^{-1}. ^{1}H NMR (300 MHz, DMSO-d_6) δ ppm: 8.77 (s, 1H), 8.71 (s, 1H), 8.46-8.49 (m, 1H), 8.31-8.34 (m, 1H), 7.91 (t, J = 9 Hz, 1H). ^{13}C NMR (75 MHz, DMSO-d_6) δ ppm: 159.7, 148, 135.9, 132.4, 131.1, 127.9, 124.8, 113.6, 112.6, 84.9.

References:

1. Rajabi, F.; Ebrahimi, A.Z.; Rabiee, A.; Pineda, A.; Luque, R. Synthesis and Characterization of Novel Pyridine Periodic Mesoporous Organosilicas and Its Catalytic Activity in the Knoevenagel Condensation Reaction. *Materials* **2020**, *13*, 1097. https://doi.org/10.3390/ma13051097.
2. Panja, S.K.; Dwivedi, N.; Saha, S. First report of the application of simple molecular complexes as organo-catalysts for Knoevenagel condensation. *RSC Adv.* **2015**, *5*, 65526–65531. https://doi.org/10.1039/C5RA09036A.
3. Khoshnavazi, R.; Bahrami, L.; Havasi, F. Organic-inorganic hybrid polyoxometalate and its graphene oxide-Fe_3O_4 nanocomposite, synthesis, characterization and their applications as nanocatalysts for the Knoevenagel condensation and the synthesis of 2,3-dihydroquinazolin-4(1H)-ones. *RSC Adv.* **2016**, *6*, 100962–100975. https://doi.org/10.1039/C6RA15339A.
